# Usage of Natural Volatile Organic Compounds as Biological Modulators of Disease

**DOI:** 10.3390/ijms22179421

**Published:** 2021-08-30

**Authors:** Min-Hee Kim, Seung-Min Lee, Ki-Wan An, Min-Jae Lee, Dae-Hun Park

**Affiliations:** 1 College of Korean Medicine, Dongshin University, Naju 58245, Korea; minhee3947@naver.com; 2 School of Veterinary Medicine, Kangwon National University, Chuncheon 24341, Korea; smin0515@gmail.com; 3 Department of Forest Resources, Chonnam National University, Gwangju 61186, Korea; kiwan@jnu.ac.kr

**Keywords:** natural volatile organic compound (NVOC), biological modulator, safety

## Abstract

Plants produce a wide variety of natural volatile organic compounds (NVOCs), many of which are unique to each species. These compounds serve many purposes, such as fending off herbivores and adapting to changes in temperature and water supply. Interestingly, although NVOCs are synthesized to deter herbivores, many of these compounds have been found to possess several therapeutic qualities, such as promoting nerve stability, enhancing sleep, and suppressing hyperresponsiveness, in addition to acting as antioxidants and anti-inflammatory agents. Therefore, many NVOCs are promising drug candidates for disease treatment and prevention. Given their volatile nature, these compounds can be administered to patients through inhalation, which is often more comfortable and convenient than other administration routes. However, the development of NVOC-based drug candidates requires a careful evaluation of the molecular mechanisms that drive their therapeutic properties to avoid potential adverse effects. Furthermore, even compounds that appear generally safe might have toxic effects depending on their dose, and therefore their toxicological assessment is also critical. In order to enhance the usage of NVOCs this short review focuses not only on the biological activities and therapeutic mode of action of representative NVOCs but also their toxic effects.

## 1. Introduction

Natural volatile organic compounds (NVOCs), also known as biogenic volatile organic compounds, are compounds that derive from living organisms such as plants. Further, volatile organic compounds (VOCs) are organic compounds that are volatile at environmental temperature; however, this definition may vary between countries and jurisdictions. For example, Canada defines VOCs as organic compounds with boiling points between 50 and 250 °C [1], whereas the European Union defines them as organic compounds with an initial boiling point less than or equal to 250 °C measured at a standard atmospheric pressure of 101.3 kPa [2]. In India [3] and the United States of America [4], these compounds tend to be defined from an environmental pollutant standpoint.

NVOCs are primarily synthesized by plants to deter herbivores [5,6] and to promote plant growth [7]. Given that the types of NVOCs produced by plants are largely species-dependent [8], plants are often phenotyped based on their unique NVOC characteristics and ratios [9]. NVOCs can be classified based on their origin (e.g., synthetic vs. natural) chemical structure (e.g., isoprenoids or terpenoids), or chemical moieties (e.g., oxygenated forms such as methanol, acetaldehyde, acetone, methyl-ethyl-ketone, or sulfurs such as furanocoumarins) [10,11]. The diversity and level of the emitted NVOCs are also determined by a variety of stimuli such as temperature and light [12,13,14], water and humidity [15,16], salt concentration [17], or the presence of ozone [18,19], among other factors.

Recent reports have discussed the many therapeutic qualities of NVOCs, including their ability to enhance sleep [20]; hypolipidemic activity and anti-cancer effect [21,22]; protective effect against viral pneumonia and anti-inflammatory effects [23]; anti-cancer and anti-oxidative effects [24,25]; neuroprotective effects [26]; anti-oxidative stress and anti-asthmatic effects [27,28,29]; alleviating effect on skin inflammation [30]; anti-*Trypanosoma* effects [31]; and industrial applications such as flavoring agents for food additives, oil for aromatherapy, commercial chemicals for many food products, soaps, and perfume [32]. However, NVOCs can also exert toxic effects, such as irritation of the pulmonary system and central nervous system [33], developmental toxicity [34,35], nephrotoxicity and hepatotoxicity [36], and allergic reactions [37,38]. Therefore, this review discusses the biological and toxicological effects of NVOCs that could be used as biological modulators of disease.

## 2. Biological Effects of Natural Volatile Compounds

### 2.1. (+)-3-Carene

**Figure 1:**
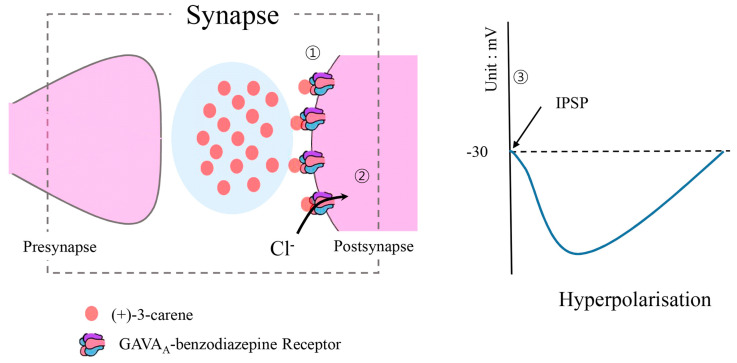
Mechanisms by which (+)-3-carene enhances sleep by stimulating the GABA_A_-benzodiazepine receptor (modified from Woo et al. 2019 [20]). ① (+)-3-carene binds on the GABA_A_-benzodiazepine receptors in the postsynaptic region. ② Cl^−^ flows into the postsynapse. ③ Inhibitory postsynaptic potential (IPSP) occurs, thus enhancing the quality and the duration of sleep under hyperpolarization. 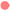: (+)-3-carene; 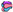: GABA_A_-benzodiazepine receptor.

(+)-3-carene is a monoterpene that is often also referred to as isodiprene, (+)-δ3-carene, δ-3-carene, and (+)-car-3-ene [39]. This compound has several biological effects such as antibacterial [40], insecticidal [41], and sleep-enhancing [20] properties. Further, Shu et al. (2019) reported that this compound induced the death of Gram-positive *Brochothrix thermosphacta* ACCC03870 and Gram-negative *Pseudomonas fluorescens* ATCC13525, which were linked to food spoilage and several diseases [42,43] via membrane breaking, metabolic dysfunction, DNA disruption, and interrupting cellular function. Insects such as the maize weevil (*Sitophilus zeamais*) can devastate entire crops, particularly grains [44]; however, (+)-3-carene has reportedly been used as an effective pest control fumigant to address this problem [41]. Further, this compound is not only an effective microbe and insect inhibitor but also enhances the quality and duration of sleep in animals by interacting with GABA_A_-benzodiazepine receptors (Figure 1) [20].

### 2.2. Camphene

**Figure 2:**
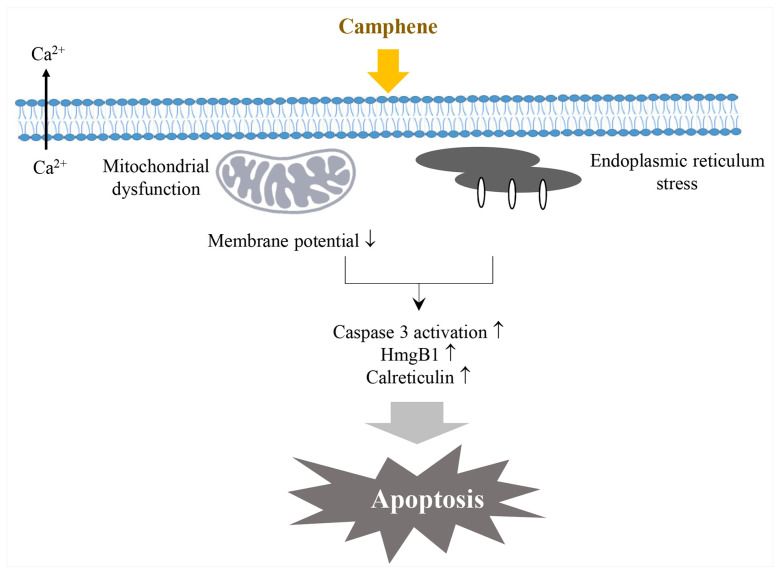
Diagram of apoptosis induction by camphene via mitochondrial dysfunction and endoplasmic reticulum stress (modified from Girola et al. 2015 [22]). Camphene stimulates Ca^2+^ efflux, downregulates membrane potential to induce mitochondrial dysfunction, induces endoplasmic reticulum stress, increases caspase 3 activation, HmgB2, and calreticulin, and finally results in apoptosis. ↑: increasing; ↓: decreasing.

Camphene is a monoterpene that is also referred to as comphene, 79–92–5, and 2,2-dimethyl-3-methylenenorbornane [45]. Camphene inhibits the growth of *Paracoccidioides lutzii*, a fungus that causes paracoccidioidomycosis [46], through protease inhibition and the dysregulation of important biological pathways [47]. Further, this compound induced the death of the old-world bollworm (*Helicoverpa armigera*) the eggs of which were dipped [48]. Regarding its therapeutic properties, this compound decreases the level of serum lipids such as cholesterol and triglycerides by upregulating sterol regulating binding protein-1 (SREBP-1) and downregulating MTP expression [21] while leaving the expression of 3-dydroxy-3-methylglutaryl coenzyme A (HMG-CoA) reductase largely unaffected [49]. Further, this compound decreased the oxidative stress on respiratory macrophages by preventing the upregulation of superoxide dismutase (SOD) and reducing glutathione (GSH) levels, in addition to decreasing the levels of lipid peroxidation and nitric oxide (NO) [50], thus stimulating tumor cell death via endoplasmic reticulum stress (Figure 2) [22].

### 2.3. Camphor

**Figure 3:**
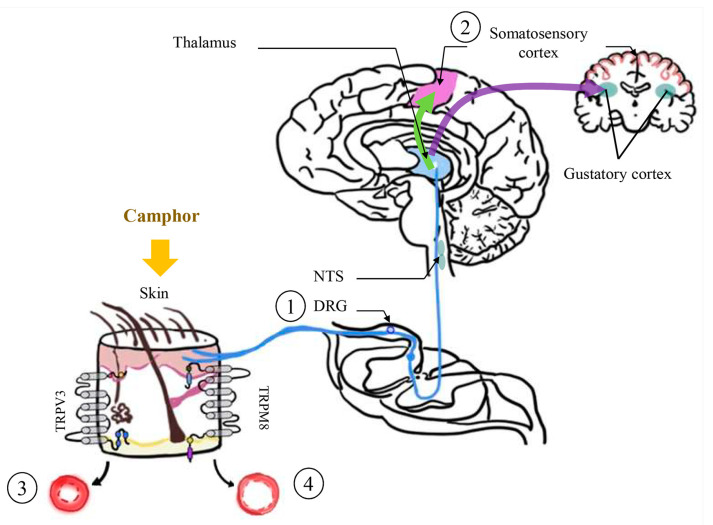
Camphor enhances cold and heat sensation via TRPM8 and TRPV3 and then induces vasoconstriction and vasodilatation (modified from Kotaka et al. 2014 [58]; Selescu et al. 2013 [59]; Steinhoff et al. 2009 [60]). When camphor is applied onto the skin, ① the stimulus affluxes via the dorsal root ganglion and nucleus tractus solitaries and ② up to the somatosensory cortex. Once the response to the stimulus is defined in the brain ③ vasoconstriction occurs if the stimulus was associated to cold temperature, otherwise ④ the blood vessels dilate if a hot temperature is sensed. DRG, dorsal root ganglion; NTS, nucleus tractus solitaries; TRPM8, transient receptor potential melastatin 8; TRPV3, transient receptor potential vanilloid 3. The blue line indicates the conduction of the nervous stimuli.

Camphor is a monoterpene that is also referred to as camphor gum, (1R)-camphor, and 464–49–3 [51]. Many tropical diseases such as malaria, dengue, and elephantiasis are caused by mosquito bites. A total of 434,000 mosquito-borne diseases were reported worldwide in 2015, thus highlighting the serious threat that these insects pose to human health [52]. Camphor has been used as a mosquito repellent in topical application, cosmetics, incense, fumigants, or sprays [53] and to inhibit the growth of some pathogenic microorganisms such as *Candida albicans*, a representative opportunistic pathogenic yeast that causes severe health complications, particularly in immunodeficient or cancer patients [54]. This compound also inhibits the growth of *Staphylococcus aureus*, a Gram-positive bacterium linked to skin abscesses and food poisoning [55], and *Pseudomonas aeruginosa*, an opportunistic Gram-negative pathogenic bacterium that infects plants and animals including humans [56] and has reportedly acquired mulita-antibiotic resistance [57]. Camphor alters cold and heat perception by modulating blood flow in the skin and muscles [58]. Particularly, cold perception is regulated by transient receptor potential melastatin 8 (TRPM8) [59], whereas heat perception is modulated by transient receptor potential vanilloid 3 (TRPV3) (Figure 3) [60].

### 2.4. 1,8-Cineol

**Figure 4:**
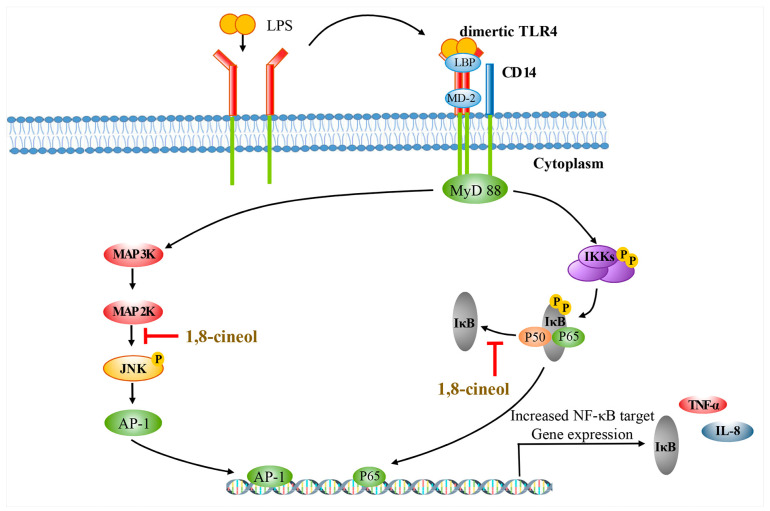
1,8-cineol downregulates LPS-induced inflammation by blocking both NF-κB translocation and JNK pathway activation (Modified from Greiner et al. 2013 [68]). 1,8-cineol prevents the separation of IκB and NF-κBp65/p55 and then blocks NF-κB translocation from the cytoplasm to the nucleus, thus acting as a transcription factor for the expression of inflammation-related cytokines such as TNF-α and IL-8. 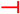: to inhibit the follow action.

1,8-cineol is a monoterpene and there are many synonyms for this compound including eucalyptol, zineol, trepan, and zedoary oil, among others [61]. In 1870, Cloez reported that 1,8-cineol accounted for 90% of the total composition of *Eucalyptus globulus* oil [62]. This compound has many therapeutic properties, such as protective effects against the influenza A virus, which induces pneumonia through cytokine modulation and the NF-κB pathway [63], as well as anti-cancer effects via G_0_/G_1_ arrest [64], anti-asthmatic effect through mucolysis, downregulation of TNF-α and IL-1β, inactivation of the NF-κB and TLR4 pathways [64,65,66,67], and anti-inflammatory and anti-oxidative effects via inhibition of NF-κB translocation and the JNK pathway (Figure 4) [68].

### 2.5. p-Cymene

**Figure 5:**
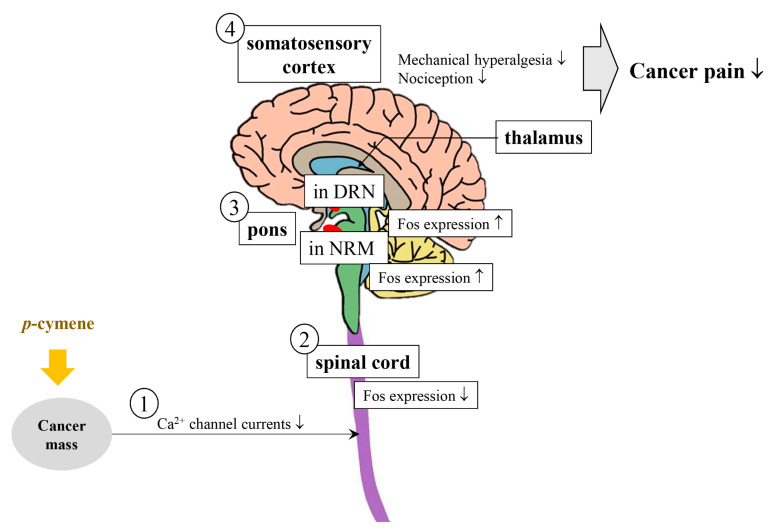
Mode of action of *p*-cymene for the reduction in cancer-associated pain via Ca^2+^ channel current downregulation, modulation of Fos expression in the spinal cord and pons, and attenuation of mechanical hyperalgesia and nociception in the somatosensory cortex (modified from Santos et al. 2019 [75]). When *p*-cymene is subcutaneously administered, ① there is a decrease in Ca^2+^ channel currents and ② the level of Fos expression decreases in the spinal cord. However, ③ Fos expression in the pons, particularly in the nucleus raphe magnus (NRM) and in the periaqueductal gray (PAG), decreases, thus resulting in ④ a decrease in both mechanical hyperalgesia and nociception through the somatosensory cortex, thus attenuating cancer-associated pain. ↑: increasing; ↓: decreasing.

There are many synonyms for *p*-cymene, including 1-isopropyl-4-methylbenzene, *p*-cimene, 4-isopropyltoluene, and camphogen [69]. This monoterpene has several biological effects, such as downregulating of both pathological Gram-positive bacteria such as *Staphylococcus aureus, Streptococcus mutans,* and *Streptococcus sanguinis*, as well as pathological Gram-negative bacteria such as *Escherichia coli* O157:H7, *Vibrio parahaemolyticus*, and *Salmonella enterica* [70]. Particularly, this compound regulates oxidative stress-induced factors such as thiobarbituric acid reactive substances (TBARS), nitrite, and catalase (CAT) activity [71]; inhibits inflammation via NF-κB pathway [72,73], promotes sleep through the GABAergic pathway [74]; and suppresses cancer proliferation and cancer-associated pain (Figure 5) [75] via apoptosis and autophagy [23].

### 2.6. Limonene

**Figure 6:**
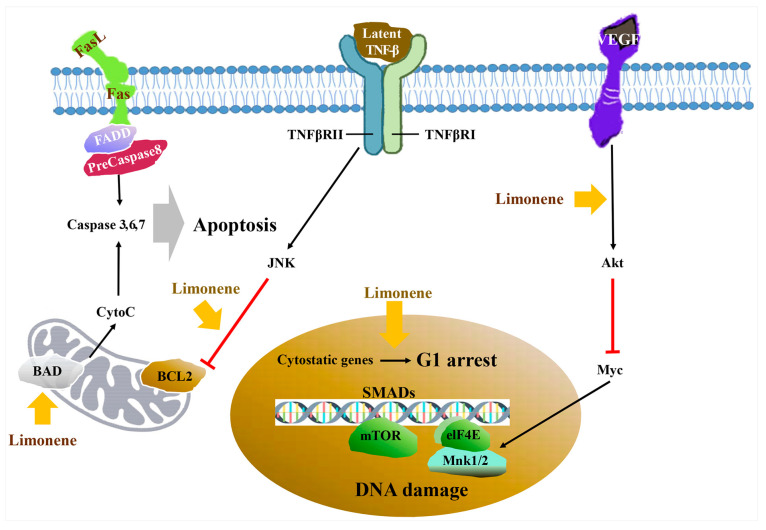
Anti-proliferation mechanism of limonene against cancer cells (modified from Shojaei et al. 2014 [80]). Limonene not only induces apoptosis by modulating the bcl-2 gene family and up-regulating pro-apoptotic proteins such as BAD and down-regulating anti-apoptotic proteins such as BCL2, but also by G1 arrest through SMAD regulation and by inhibiting metastasis through suppression of Myc caused by the vascular endothelial growth factor (VEGF) receptor/Akt pathway. 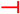: to inhibit the follow action.

Limonene is another monoterpene whose synonyms include eulimen, dipentene, nesol, goldflush II, cajeputene, and dipanol [76]. This compound effectively controls *Listeria monocytogenes*, a food poisoning-associated microbe, by disrupting its cell membrane and decreasing ATP activity [77]. Further, it prevents Aβ42-induced neurotoxicity in a *Drosophila* Alzheimer’s disease model by eliminating H_2_O_2_ and nitric oxide-induced inflammation and cell death [26]. This compound also prevents reactive oxygen species (ROS)-induced gastritis by both downregulating proinflammatory cytokines such as TNF-α, IL-1β, and IL-6 and upregulating the anti-inflammatory cytokine IL-10 [78]. Limonene has also been reported to modulate depressive behaviors by suppressing psychostimulant and monoamine neurotransmitters, in addition to inhibiting both neurotrophic factor release and its receptor activation [79], as well as the proliferation of cancer cells via the stimulation of apoptosis (Figure 6) [80].

### 2.7. Linalool

**Figure 7:**
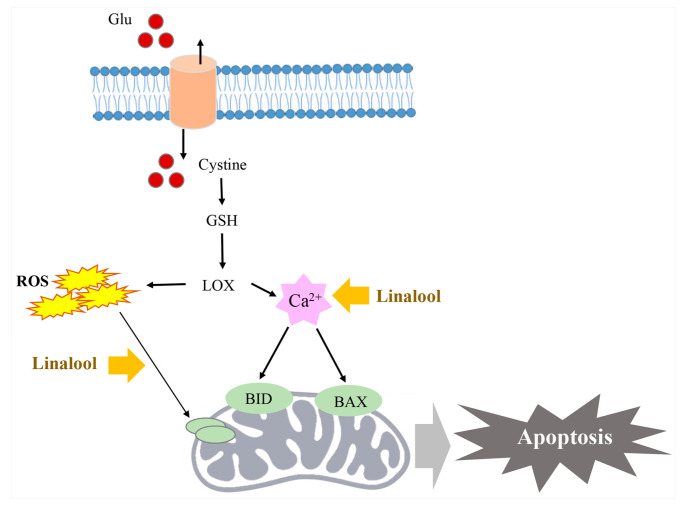
Linalool decreases oxidative stress-induced cell death by regulating glutamate metabolism in the hippocampus (modified from Sabogal-Guaqueta et al. 2019 [27]). In several neurological conditions such as Alzheimer’s disease and neuroinflammation, glutamate induces cysteine influx into the cytoplasm and stimulates oxidative stress, thus inducing apoptosis. Linalool prevents ROS generation and Ca^2+^ activation in the mitochondria in the cornu ammonis 1 and 3 and dentate gyrus in the hippocampal regions of the brain.

Linalool is a monoterpene that is also widely known as coriandrol, howood oil, allo-ocimenol, caswell No. 526A, and phantol [81]. It has antifungal effects against dermatophytes such as *Microsporum* spp. and *Trichophyton* spp. and has synergic effects when combined with azole [82]. Moreover, this compound promotes operant behavior through the GABA receptor [83], is used as an ingredient for perfume and cosmetics with anti-oxidative effects and low cytotoxicity [84], prevents ovalbumin-induced asthma occurrence by inhibiting airway remodeling [85], and decreases oxidative stress-induced cell death by regulating glutamate metabolism in the cornu ammonis 1 and 3 and dentate gyrus in the hippocampal regions of the brain (Figure 7) [27].

### 2.8. Myrcene

**Figure 8:**
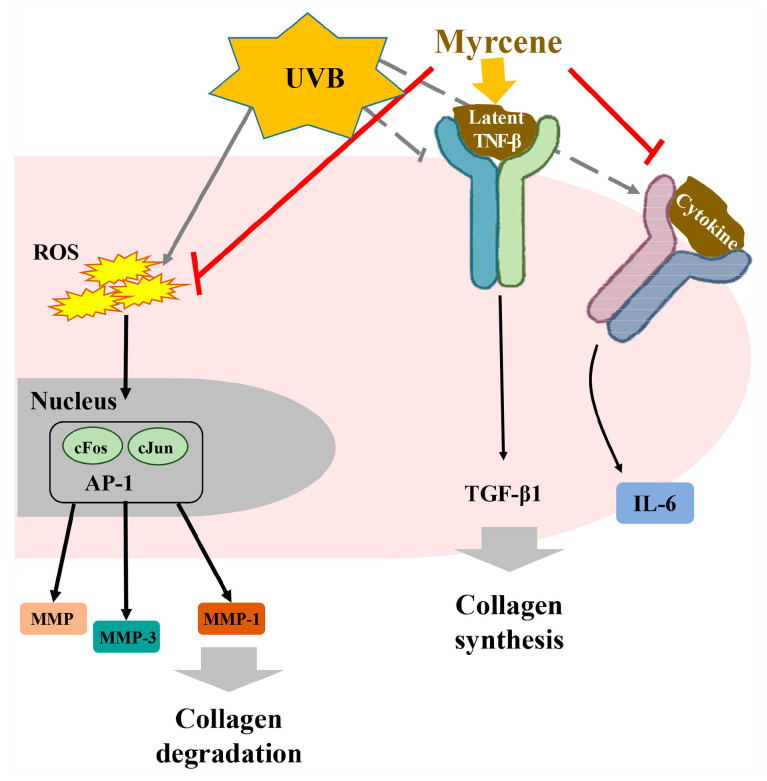
Myrcene effectively controls UVB-radiated skin aging through MMPs and IL-6 downregulation and collagen synthesis through TGF-β upregulation (Modified from Hwang 2017 [80]). Myrcene not only blocks collagen degradation by protecting UVB-induced ROS but also stimulates collagen synthesis through TGF-β1 activation. UVB-induced ROS finally induces collagen degradation, which is caused by MMP-1, MMP-3, and MMP-9 that are made by transcription factors such as AP-1, cFos, and cJun. UVB radiation inhibits TGF-β1 activation, which stimulates collagen synthesis. 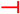: to inhibit the follow action.

Myrcene is a monoterpene that is also known as MFCD00008908, FEMA No. 2762, CCRIS 3725, β-geraniolene, and β-mircene [86]. It controls cystic echinococcosis via morphological alteration in *Echinococcus granulosus* larval cells [87], attenuates pain through TRPV1 regulation [88], prevents ovalbumin-induced neonatal asthma by controlling pulmonary matrix changes [29], inhibits glucocorticoid malfunction-induced renal impairment, which is caused by oxidative stress and inflammation [28], protects against MMP synthesis caused by UVB-induced ROS and IL-6 expression, and reverts UVB-induced TGF-β1 down-regulation (Figure 8) [89].

### 2.9. α-Phellandrene

**Figure 9:**
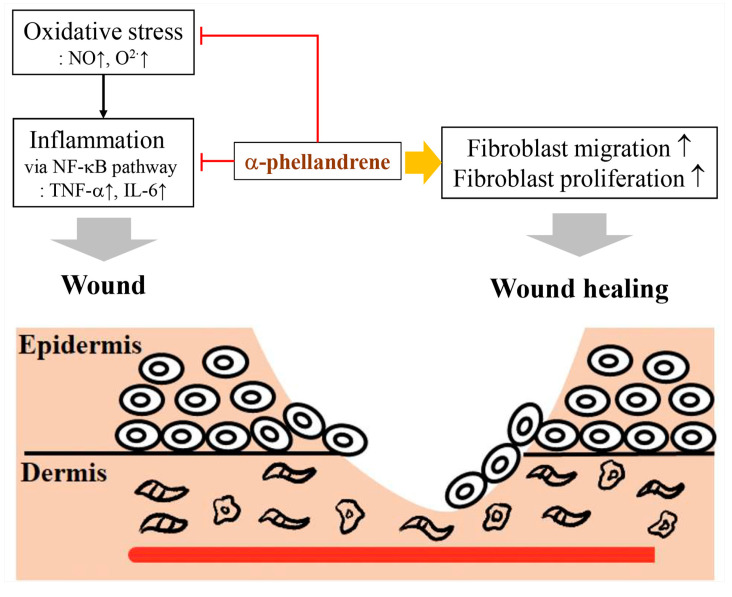
α-phellandrene promotes wound healing by inhibiting oxidative stress and inflammation, in addition to stimulating wound healing via potent fibroblast activation (modified from de Christo Scherer et al. 2019 [93]). ↑: increasing; 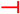: to inhibit the follow action.

α-phellandrene is a monoterpene that is also known as ZINC8418983, *p*-mentha-1,5-diene, (5S)-5-isopropyl-2-methylcyclohexa-1,3-diene, CHEB:367, and Q25933668 [90]. α-phellandrene has been used to control insect pests on larvae under dipping or on adults under topical application such as the southern house mosquito (*Culex quinquefasciatus*), the African cotton leafworm (*Spodoptera littoralis*), and the common housefly (*Musca domestica*) [91]. This compound also inhibits the proliferation of bacteria such as *Staphylococcus pneumoniae, Vibrio cholerae,* and *Escherichia coli*, as well as fungi such as *Fusarium moniliforme* [92], and promotes the recovery of skin wounds by reducing oxidative stress-induced inflammation and stimulating fibroblast activity (e.g., migration and proliferation) (Figure 9) [93].

### 2.10. Pinene

**Figure 10:**
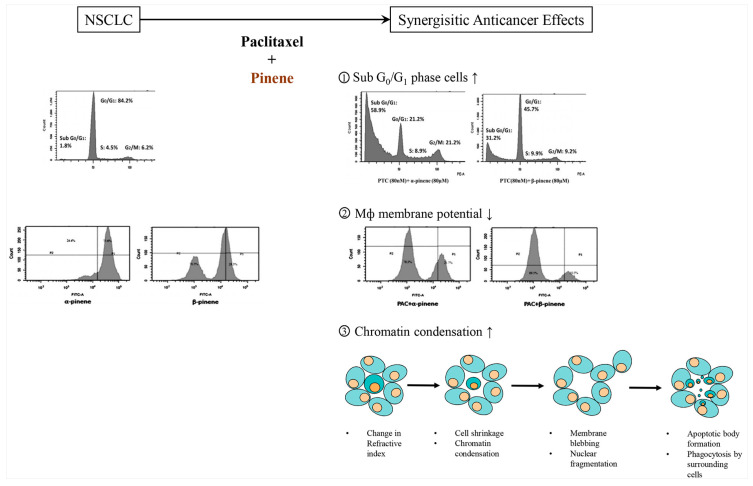
α- and β-pinene have synergistic anticancer effects against non-small cell lung carcinoma (NSCLC) when co-administered with paclitaxel (Modified from Zhang et al. 2019 [79]). ↑: increasing; ↓: decreasing.

Pinene consists of two isomers including α-pinene and β-pinene [94]. α-pinene and β-pinene have many therapeutic properties such as antimicrobial activity, anti-proliferation effects against cancer cells, antioxidation, and anti-inflammation [95], in addition to gastroprotective activity by modulating gastrointestinal transitional time [96], as well as synergistic effects against the proliferation of non-small cell lung carcinoma (NSCLC) when co-administered with paclitaxel (Figure 10) [97].

#### 2.10.1. α-Pinene

α-pinene has many therapeutic properties, such as antiviral effects against herpes simplex virus type 1 (HSV-1) [98], modulation of antibiotic resistance in *Campylobacter jejuni* through antibiotic efflux down-regulation [99], apoptosis induction in cancer cells via ROS production, mitochondrial malfunction, caspase cascade activation, and inhibition of metastasis [100]. Additionally, this compound mediates sleep-enhancement-induced hypnosis through GABA_A_-benzodiazepine receptor modulation [101], and preventive and therapeutic effects against allergic rhinitis via the regulation of disease-related factors such as IgE, IL-4, NF-κB, and receptor-interacting protein 2 (RIP2), as well as eosinophils infiltration in the lungs [102].

#### 2.10.2. β-Pinene

Although the chemical formula of β-pinene is the same as that of its isomer α-pinene, the former possesses unique biological properties. For instance, β-pinene attenuates Cr-induced phytotoxicity due to its antioxidant properties [103] and possesses antifungal and anti-biofilm properties against *Candida spp.* [104]. This compound also suppresses hypertension via Ca^2+^ influx inhibition-mediated vasorelaxation [105] and has antiviral effects against herpes simplex virus type 1 (HSV-1) [106].

### 2.11. α-Terpinen

**Figure 11:**
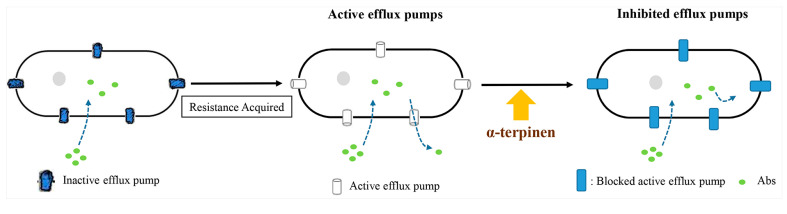
α-terpinen blocks the antibiotic-resistance of *Staphylococcus aureus* through NorA efflux pump inactivation (modified from de Morais Oliveira-Tintino et al. 2018 [109]).

α-terpinen is a monoterpene that is also known as *p*-mentha-1,3-diene, terpilene, FEMA No. 3558, CCRIS 9058, Tox21_301126, and ZINC967593 [107]. This compound has anti-parasitic activity against *Trypanosoma evansi* when used in oral treatment or intraperitoneal injection and increases the life expectancy of infected animals [31]. Similar to α-pinene, α-terpinen has antiviral effects against HSV-1 by decreasing infection rate and selectivity to the virus [106]. Additionally, this compound has antispasmodic activity in the trachea [108] and blocks antibiotic-resistance in *Staphylococcus aureus* through NorA efflux-pump inactivation (Figure 11) [109].

### 2.12. Terpinolene

**Figure 12:**
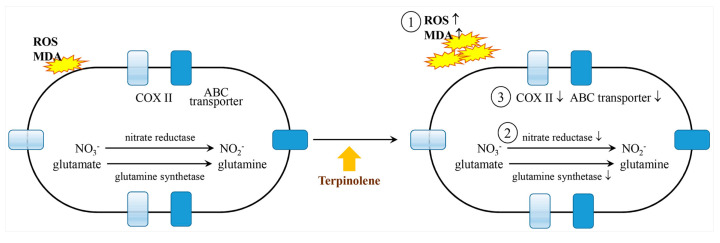
Terpinolene blocks the propagation of *Microcystis aeruginosa* through ① ROS and MDA-induced photosynthetic inactivity, ② inhibiting the activity of nitrate reductase and glutamine synthetase, and ③ increasing the efficacies of ABC transporters and COX II in the plasma membrane (modified from Zhao et al. 2020 [115]). Reactive oxygen species, ROS; malondialdehyde, MDA; nitrate reductase, NR; glutamine synthetase, GS; ATP-binding cassette transporters, ABC transporter; cytochrome c oxidase subunit II, COX II. ↑: increasing; ↓: decreasing

The monoterpene terpinolene is also known as tereben, *p*-Menth-1,4(8)-diene, isoterpinene, Nofmer TP, and 1,4(8)-terpadiene [110]. Terpinolene makes *Staphylococcus aureus* more susceptible to antibiotics by inactivating the resistance-mediated quaternary ammonium compounds C (QacC) efflux pump and β-lactamases [111]. Additionally, this compound has wound-healing effects [93], acts as a relaxant when inhaled [112], decelerates brain tumor growth and oxidative stress [25], controls the proliferation of *Microcystis aeruginosa* through (1) upregulation of reactive oxygen species (ROS) and malondialdehyde (MDA) to activate photosynthesis, (2) inhibition of the activities of important biological enzymes, e.g., nitrate reductase (NR) for protein synthesis and glutamine synthetase (GS) for the production of glutamate, and (3) upregulation of ATP-binding cassette transporters (ABC transporter), which are induced by ions and xenobiotics [113], as well as cytochrome c oxidase subunit II (COX II), which participates in ATP regulation [114] in the plasma membrane (Figure 12) [115].

## 3. NVOC Safety

Despite the promising health-promoting properties of NVOCs, their safety must be thoroughly assessed prior to their implementation as pharmaceutical candidates or dietary supplements. NVOCs are used in a variety of products, such as cosmetic materials (e.g., soap, perfume, lotion), flavor agents, aromatherapy, additives in several food products (i.e., baked goods, frozen dairy, gelatins), and vector repellents [20,22,23,24,25,26,27,28,31,41,62,84,89]; however, their safe use is highly regulated. NVOCs can induce toxic effects depending on their dose and, therefore, toxicology studies are critical (Table 1). (+)-3-carene irritates the lungs and central nervous system (CNS) [33], camphor irritates the gastrointestinal tract and CNS [116], and 1,8-cineol induces genotoxicity via oxidative DNA damage [35]. *p*-cymene is thought to be less toxic, but it induces neurochemical abnormalities after four weeks of inhalation [117]. Limonene has been found to induce hepatotoxicity and neurotoxicity [36] and oxidized forms of limonene and linalool induce allergic reactions in the skin [37]. The toxicity of myrcene has long been the topic of much debate due to its potential genotoxicity or nephrotoxicity and, therefore, the US FDA prohibited its use as a food additive [118]. Further, oxidized α-phellandrene and oxidized terpinolene induce contact allergy [38], α-pinene induces pulmonary inflammation [119], β-pinene can irritate the skin and mucous systems [120], whereas α-terpinene has embryo/fetotoxic effects [121]. In order to use NVOCs as pharmaceutical candidates or dietary supplements, we should use them in quantities the enormous volume needs. Then, the level of them in the forest should be analyzed and they should be collected from the air or from the extracts. All of the 12 NVOCs which are discussed in this review could be analyzed by gas chromatography-mass spectrometry (GC-MS) based on their biological characters [122,123,124,125].

## 4. Perspectives

NVOCs are promising modulators of disease, as each NVOC has a potency to control diseases such as insomnia via sleep-enhancing by (+)-3-carene [20], cancer proliferation via induction of cancer cells’ apoptosis by camphene [22], microbial infection by camphor [54], chronic disease through suppression of oxidative stress and inflammation and asthma/COPD via modulation of NF-κB activation and TNF-α secretion by 1,8-cineol [64,65,66,67], cancer proliferation via cancer cells’ death and cancer pain by p-cymene [75], mental disorder via antidepressant-like effects of limonene [79], allergy through controlling MAPKs/NF-κB pathway by linalool [85], pain via TRPV1 regulation by myrcene [88], skin damage through controlling inflammation by α-phellandrene [93], allergic rhinitis through NF-κB controlling and caspase pathway by α-pinene [100], hypertension via modulation of Ca2+ influx by β-pinene [105], antibiotic-resistance of Staphylococcus aureus through NorA efflux pump inactivation by α-terpinene [109], and Microcystis aeruginosa propagation through induction oxidative stress and carbonyl stress by terpinolene [115]. However, characterizing the mechanisms by which NVOCs inhibit disease progression is critical for their wide application in clinical contexts. NVOCs can be administered either directly (e.g., oral administration, inhalation) or indirectly using a syringe, nebulizer, or patch. However, oral administration and inhalation are more convenient to the patients and caregivers (e.g., nurses, doctors). Most NVOCs have relatively low boiling points, thus facilitating their administration via inhalation. This is an important advantage, as it enables the administration of therapeutic compounds without the need for highly specialized equipment.

Nevertheless, the safety of NVOCs must also be carefully evaluated to ensure their safety. Paracelsus [126] is credited for stating that “the dose makes the poison,” meaning that virtually all compounds can be toxic if administered at high enough doses.

Currently, most drugs and therapeutic compounds can be chemically synthesized [127], and NVOCs are no exception. However, artificially synthesized and natural NVOCs could have different physiological effects. For example, myrcene has been long used as a food additive and a flavoring agent or adjuvant due to its pleasant aroma; however, its safety has been debated for several years. In 2010 the National Toxicology Program (NTP) reported that β-myrcene could induce renal carcinogenesis in mice and rats [128]. Nevertheless, the FDA ultimately determined that β-myrcene did not induce genotoxicity. Moreover, although this compound was linked to renal carcinogenesis in laboratory animals, some speculated that the compound was safe for human use as a synthetic food additive. Nevertheless, the FDA prohibited the use of synthetic β-myrcene as a food additive in 2018 [129]. This case serves as a precedent for the use of NVOCs (particularly those of natural origin) in medications and food products. Nevertheless, the current changes in climate conditions could induce an increase in NVOC concentrations in plants as a stress response mechanism, which might increase the likelihood of toxic effects. Therefore, future studies should focus on the dose-dependent toxicity of NVOCs.

## Figures and Tables

**Table 1:** Toxicological effects of natural volatile organic compounds (NVOCs).

Natural Volatile Organic Compound	Chemical Structure	Molecular Weight (g/mol)	Toxicological Effect	References
(+)-3-carene	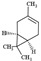	136.24	To irritate pulmonary system and central nervous system	[33]
Camphene	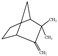	136.23	To irritate serious eye	[45]
Camphor	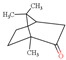	152.23	To irritate gastrointestinal tract and central nervous system	[116]
1,8-cineol	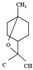	154.25	Genotoxicity via inducing oxidative DNA damage	[35]
*p*-cymene	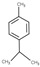	134.22	To induce neurochemical problem by 4 weeks of inhalation	[117]
Limonene	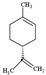	136.23	Hepatotoxicity and neurotoxicity Oxidized form makes skin allergy.	[36,37]
Linalool	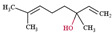	154.25	To induce skin allergy by oxidized linalool	[37]
Myrcene	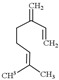	136.23	It does not have unique toxicity but in 2018 US FDA decided to stop the usage of it as a food additive.	[118]
ɑ-phellandrene	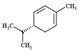	136.20	To make contact allergy	[38]
ɑ-pinene	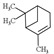	136.23	To induce pulmonary inflammation	[119]
ß-pinene	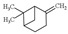	136.23	To irritate skin and mucous system	[120]
ɑ-terpinene	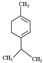	136.23	Embryofoetal toxicity	[119]
Terpinolene	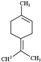	136.23	Oxidized forms can make allergic contact dermatitis.	[38]
